# Researching trends on stroke: bibliometric analysis of the highly cited papers

**DOI:** 10.3389/fstro.2024.1447369

**Published:** 2024-11-07

**Authors:** Li Fan, Fuyan Shi, Suzhen Wang

**Affiliations:** ^1^Department of Health Statistics, School of Public Health, Shandong Second Medical University, Weifang, Shandong, China; ^2^People's Hospital of Ningxia Hui Autonomous Region, Yinchuan, Ningxia, China

**Keywords:** stroke, highly cited, bibliometrics, CiteSpace, VOSviewer

## Abstract

This study assessed the progression of stroke using bibliometric methods. By March 31, 2024, highly cited papers on stroke were collected from the Web of Science Core Collection. Collaboration and keyword conjunction analyses, along with their visual representations, were conducted using VOSviewer. CiteSpace was employed to identify keywords and reference sources. A comprehensive review of 2,509 highly cited studies on stroke was conducted. Notably, the United States, China, and the United Kingdom have emerged as leading contributors to this research domain, with the New England Journal of Medicine having the highest number of publications. YUSUF S had the highest H-index among all authors. The key terms frequently encountered were “stroke,” “atrial fibrillation,” and “cardiovascular disease.” This study performed a detailed bibliometric review of stroke research over the past decade, shedding light on the participation of various countries, organizations, authors, journals, and articles in the field. These insights offer a broad snapshot of the global stroke research trends.

## 1 Introduction

Owing to the high frequency, prevalence, disability rate, and mortality rate of stroke, it has received widespread attention from the global research community (GBD 2019 Stroke Collaborators, 2021). There is a large amount of scholarly literature on stroke, and numerous studies have been published in leading medical journals. Many researchers have conducted comprehensive meta-analyses to examine the various factors related to stroke. However, systematic bibliometric examinations providing a comprehensive view of publications, countries, research institutions, journals, authors, and keywords in published formats are lacking. This bibliometric study aims to fill this gap by providing a detailed and insightful overview of the existing knowledge in stroke research. Bibliometrics is an analytical discipline that uses quantitative and statistical methods to study the production and dissemination of scholarly literature (Hicks et al., 2015). It involves careful collection, organization, and analysis of bibliographic data such as citation counts, co-authorship networks, and publication venues (Mukherjee et al., 2022). The advantages of bibliometrics include their ability to quantify and identify the impact of research, provide evidence-based evaluations of scientific output, and track the progress and influence of research over time. It also helps to identify emerging trends, developing fields, collaborative efforts, and guide strategic planning and resource allocation within research institutions (Jiang et al., 2023). With the expanding volume of scientific literature and growing importance of research impact, the role of bibliometrics in evaluating and interpreting research has become increasingly important.

This study used a comprehensive bibliometric analysis to assess the trajectory, breakthroughs, and key issues within the existing stroke research. By summarizing and analyzing current findings and trends, this study fills critical gaps in the existing literature, providing researchers, clinicians, epidemiologists, and policymakers with a refined and comprehensive perspective on the current state of stroke research.

## 2 Materials and methods

### 2.1 Data retrieval strategies

We used the Web of Science Core Collection (WoSCC), the most authoritative and comprehensive global science database, to search for stroke-related literature. The search spanned articles uploaded to the database on March 31, 2024. The search terms were as follows: ((((((TS = (stroke)) OR TS = (cerebral infarction)) OR TS = (ischemic stroke)) OR TS = (intracerebral hemorrhage)) OR TS = (hemorrhagic stroke)) OR TS = (subarachnoid hemorrhage)). Because of the absence of animal testing or experimental protocols in our study, ethical clearance was not required. Our selection criteria were confined to Highly Cited Papers with document types of “article” or “review” in the English language, targeting a specific subject matter and research objective, and ensuring uniformity in language for subsequent analysis. All other literature types and non-English articles were excluded from the review. We conducted searches and examined all articles retrieved in various formats on the same day in plain text form to create master files for use with different bibliometric tools (Yeung, 2019). Thereafter, we extracted essential information such as author names, source of study, title, keywords, and cited references from the exported articles to mitigate potential errors during retrieval at different instances.

### 2.2 Bibliometric analysis

In this study, we employed R version 4.3.3 (Ihaka and Gentleman, 1996), VOSviewer (Van Eck and Waltman, 2010), and CiteSpace (Chen, 2006) to conduct bibliometric analysis. We utilized the Bibliometrix R package version 4.3.3 to calculate the frequency of international collaboration among countries (Aria and Cuccurullo, 2017). VOSviewer was used to determine the numbers of publications, citations, and keywords. The built-in clustering algorithm of the software enabled the construction and visualization of co-occurrence networks of key terms from scientific literature (Jiang et al., 2022). Our main focus was on co-authorship and co-occurrence analysis, which helped us to understand the collaboration between countries, institutions, and authors.

We used CiteSpace to identify highly cited references and keywords that had witnessed substantial citation growth over a specific period. Using online bibliometrics, we visualized international collaborations between countries. We analyzed the annual scientific output and average citations per year using Microsoft Excel.

## 3 Results

### 3.1 Overview of publication status

**Figure 1:**
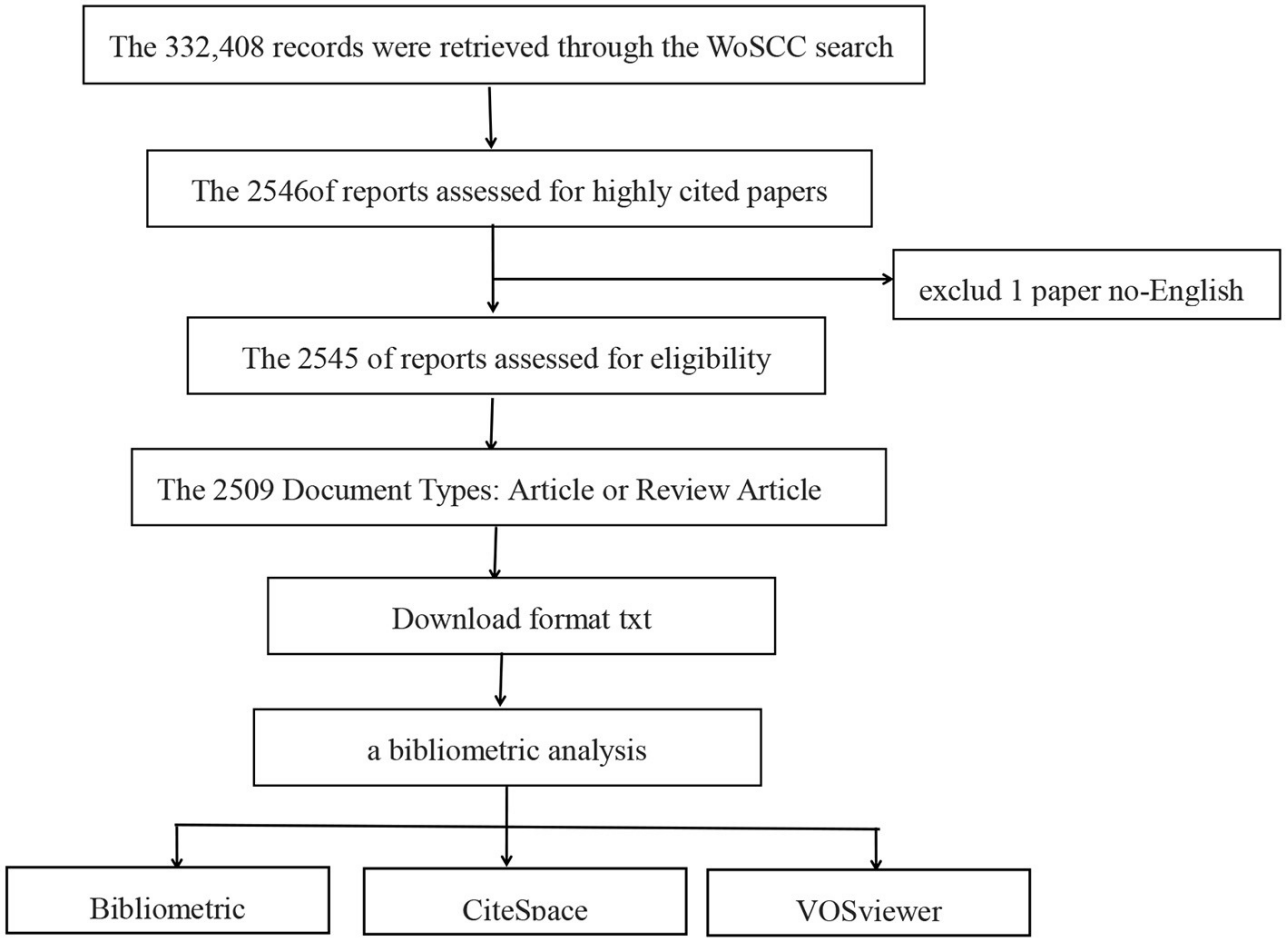
Flow-chart of the study.

**Figure 2:**
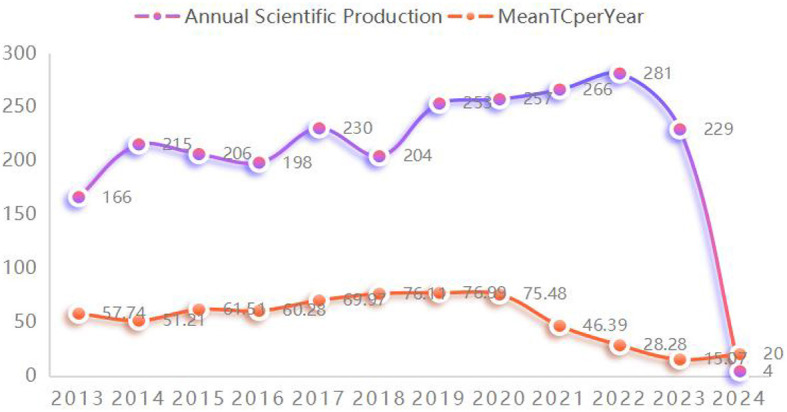
Annual scientific production and average citations per year.

From the extensive collection of 332,408 research studies, a selected group of 2,509 publications that had been extensively referenced was identified for closer analysis. This collection includes 1,749 articles and 760 reviews, as depicted in Figure 1. Figure 2 illustrates that the most recent of these highly cited papers on stroke were released in 2024, with the earliest being from 2013. A review of the most highly cited periodicals revealed that the annual volume of scientific contributions will reach its zenith in 2022. In addition, 2020 saw the highest annual average number of citations per paper.

### 3.2 Analysis of national publication counts

**Figure 3:**
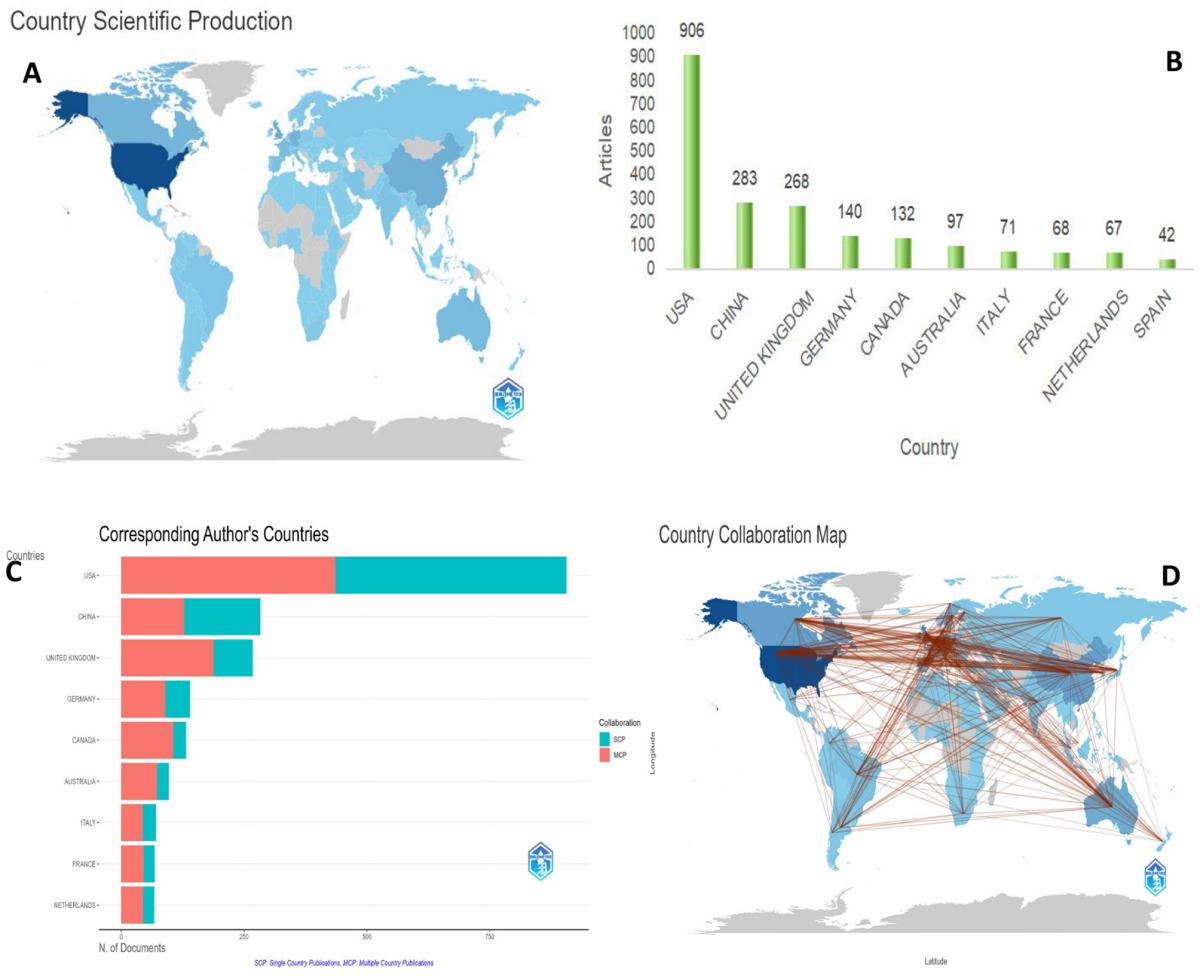
**(A)** Country scientific production; **(B)** the output of top 10 countries; **(C)** the corresponding author's countries; **(D)** the country collaboration map.

To explore the distribution of research output across countries and regions, a comprehensive analysis of national publication tallies was conducted (Figures 3A, B). The dataset reviewed included research contributions from 113 countries or regions and more than 5,186 distinct institutions. As depicted in Figure 3, the United States emerged as the leading contributor, with 906 published works, followed by China (283), the United Kingdom (268), Germany (140), and Canada (132). All other countries or regions included in the analysis had a total publication count of < 100.

In addition to the overall output analysis, we mapped international collaborations in the field of stroke research, as shown in Figure 3D. The findings revealed that the United States is at the forefront of stroke research collaborations. There is a high degree of global cooperation, particularly among the developed nations in Europe and North America (Figure 3C). The top 10 countries in terms of collaboration, the top 10 countries had a median country-to-country partnership (MCP) ratio exceeding 45% when collaborating with international authors. The United States collaborated the most in the United Kingdom (420 times), Canada (365 times), and Germany (323 times). In the United Kingdom, the most frequent collaborative partners were Germany (242 times) and Canada (241 times).

### 3.3 Analysis of institution publications

**Figure 4:**
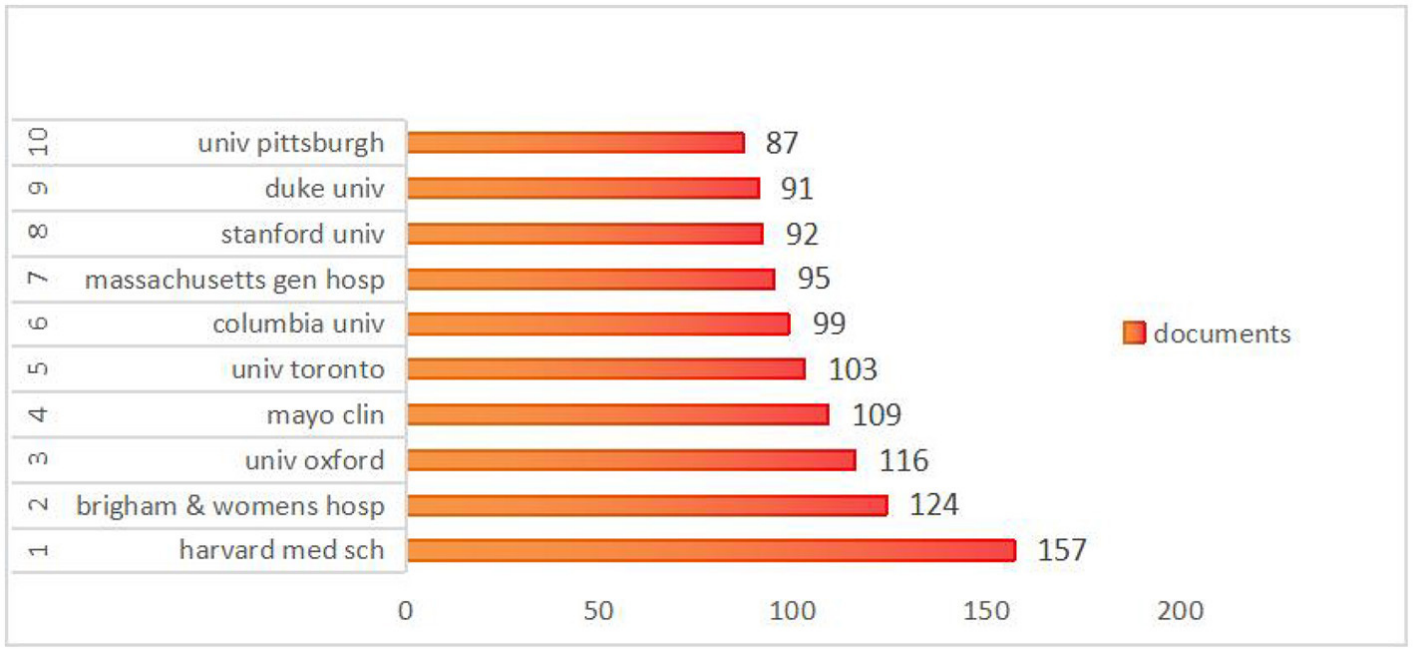
The top 10 institutions with the most publications in the field of stroke.

**Figure 5:**
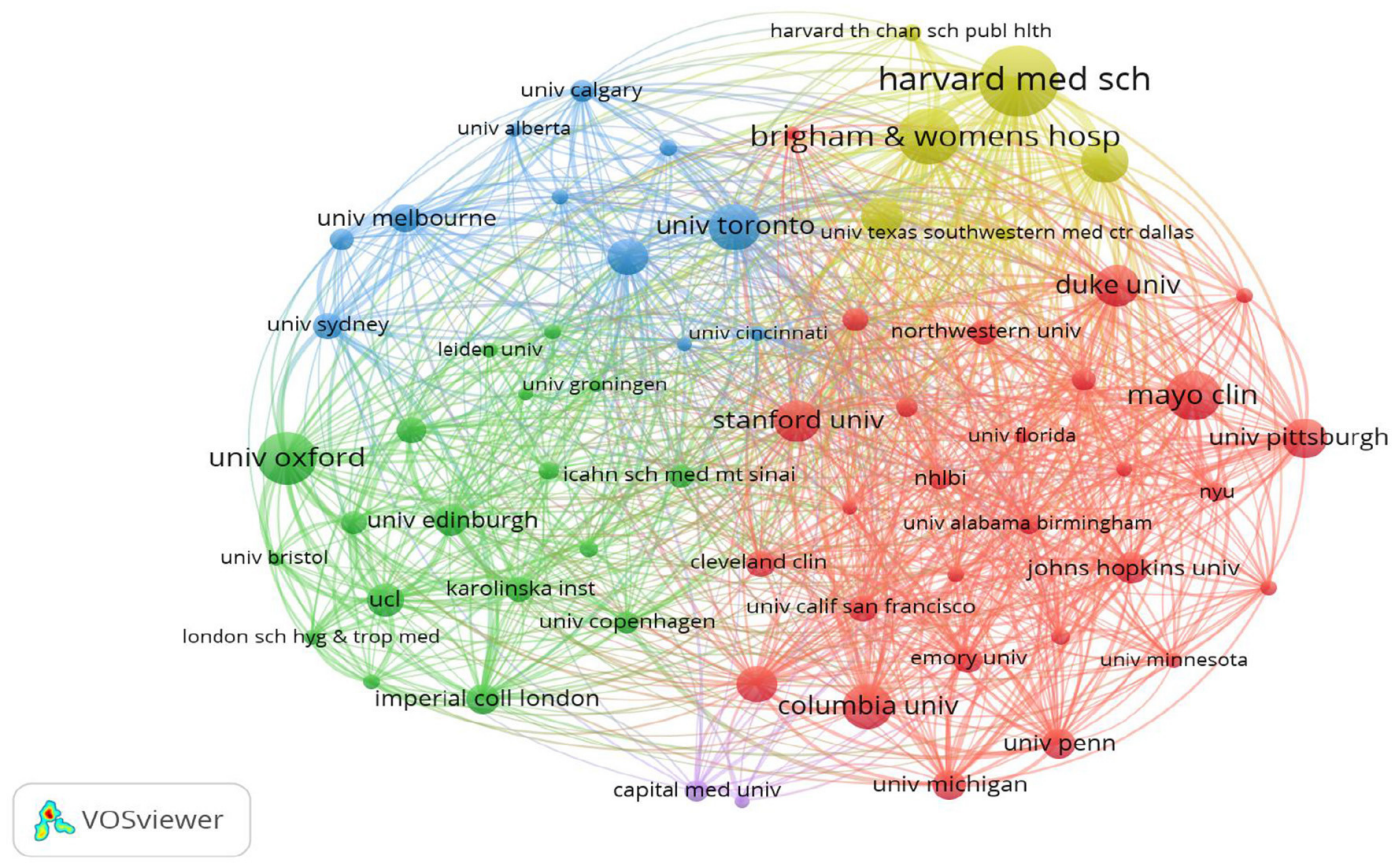
Research institute co-occurrence network.

An analysis was conducted to examine the contributions of various institutions to the domain of stroke research, which revealed the publication output of nearly 5,186 institutions globally. As depicted in Figure 4, the five leading institutions published more than 100 papers, with Harvard Medical School emerging as the leader with 157 publications. Brigham and Women's Hospital closely followed the 124 publications. Additionally, to delve deeper into collaborative efforts between institutions, a co-authorship analysis was conducted across all published papers. As shown in Figure 5, 68 institutions had published at least 25 papers. These 85 institutions were clustered into four groups, with the red cluster being the most prominent, comprising 36 members, primarily from the USA. The green cluster was the second-largest cluster, consisting of 31 institutions. The third cluster is the blue cluster with nine institutions, and the smallest is the yellow cluster, which consists of eight institutions.

### 3.4 Analysis of publication quantity and journal impact

**Figure 6:**
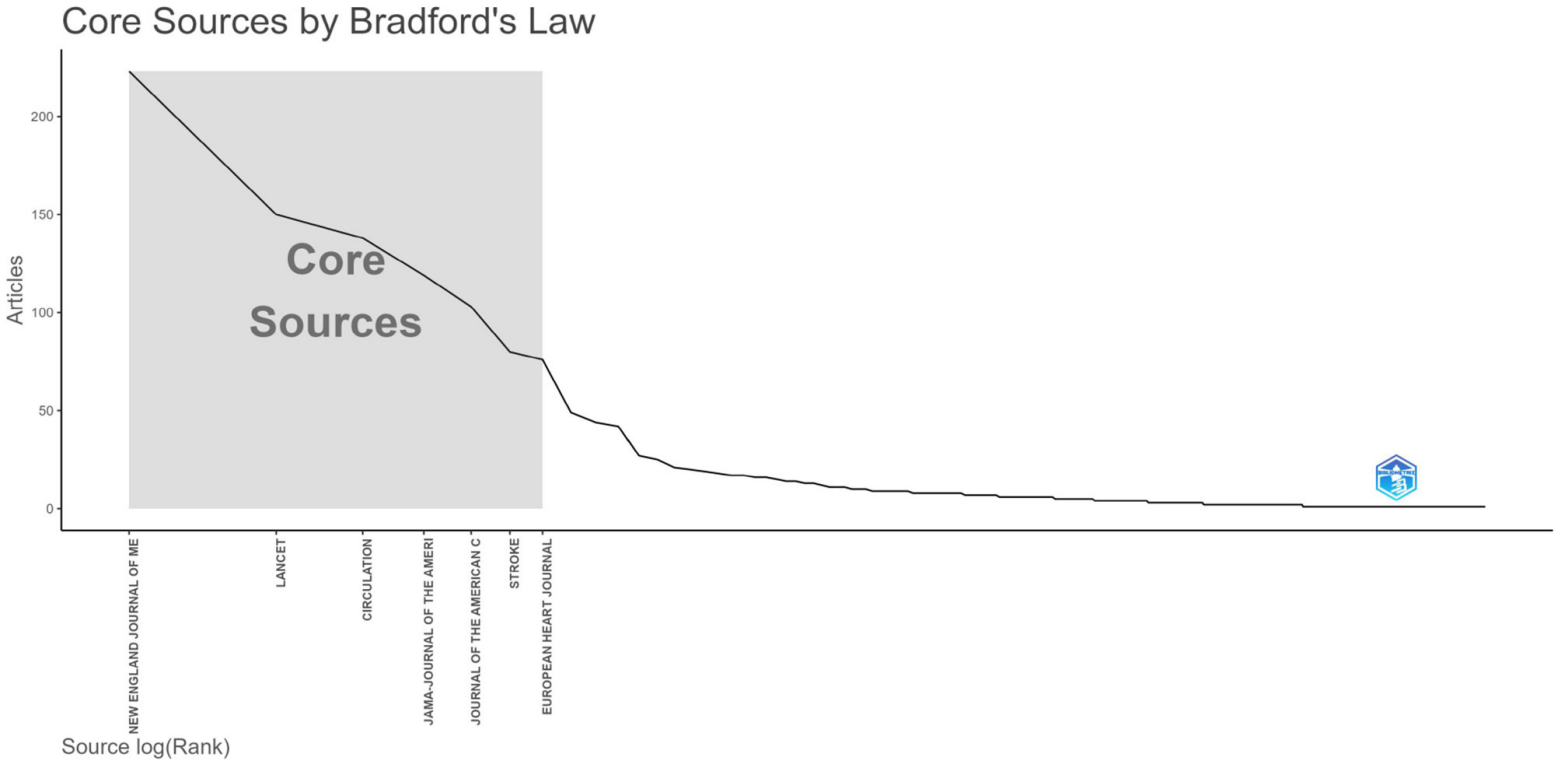
Core sources by bradford's law in field of stroke.

This study included 2,509 articles published in 590 journals. Table 1 lists the top 10 journals ranked by publication quantity and latest 2022 impact factors (IF). The top 10 journals were all top journals, with an average impact factor of 73.13, and four journals had an influence factor of more than 100. All top 10 journals were in the first quartile (Q1) of the Journal Citation Reports (JCR). As shown in Figure 6, we can see from the graph of Bradford's dispersion law that the core journals in the research field include the top seven journals in terms of their publications.

**Table 1:** Top 10 journals in the field of stroke.

**Rank**	**Source**	**Article**	**Country**	**IF**	**H-index**	**JCR-c**
1	New England Journal of Medicine	223	US	158.50	185	Q1
2	Lancet	150	UK	168.90	128	Q1
3	Circulation	138	US	37.80	109	Q1
4	JAMA-Journal of the American Medical Association	119	US	120.70	98	Q1
5	Journal of the American College of Cardiology	103	US	24.00	87	Q1
6	Stroke	80	US	8.30	62	Q1
7	European Heart Journal	76	UK	39.30	69	Q1
8	Lancet Neurology	49	UK	48.00	44	Q1
9	Circulation Research	44	US	20.10	42	Q1
10	BMJ-British Medical Journal	42	US	105.70	40	Q1

### 3.5 Author impact analysis

A comprehensive analysis of 15,324 contributors to seminal stroke studies revealed that YUSUF S led the pack with 43 published studies and an H-index of 43. Second, GUPTA R and LIP GYH also deserve mention, producing 40 articles each, with an H-index of 39 (Table 2).

**Table 2:** Top 10 authors in the highly cited papers in the field of stroke research.

**Rank**	**Authors**	**Articles**	***h*_index**
1	Yusuf S	43	43
2	Gupta R	40	39
3	Lip GYH	40	39
4	Bhatt DL	39	36
5	Steg PG	30	29
6	Mcguire DK	29	28
7	Leon MB	28	24
8	Zhang Y	28	26
9	Diaz R	27	27
10	Connolly SJ	25	25

**Figure 7:**
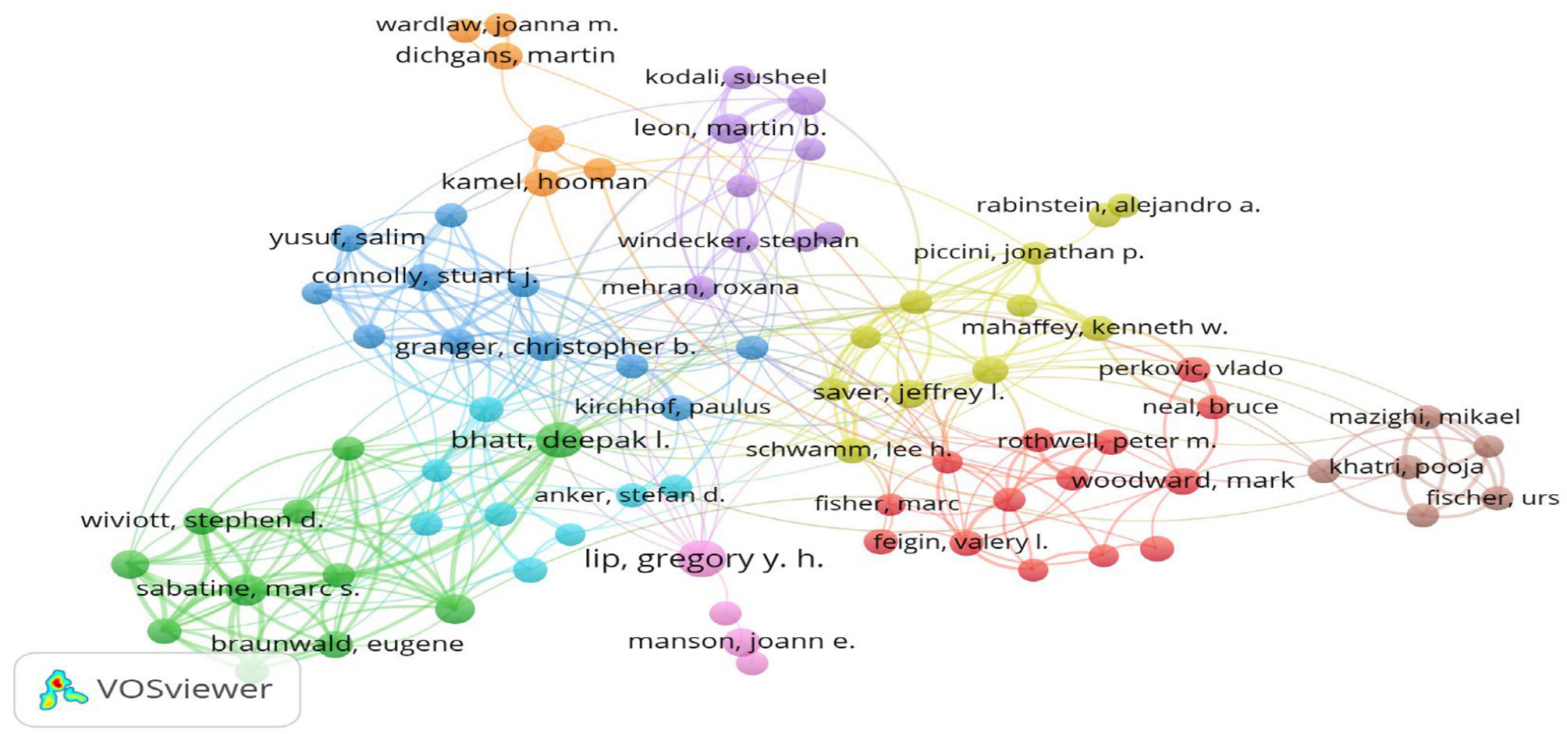
Authors in stroke researches visualization.

The collaborative networks among these researchers are depicted in Figure 7, where the size of the nodes corresponds to the number of articles authored, and the color coding denotes the clusters. Eighty authors, each having published eight or more articles, were identified and organized into nine clusters. These clusters exhibit mutually cooperative patterns. The largest cluster comprised 14 research groups, whereas the smallest contained only four research groups.

### 3.6 Co-cited references

**Figure 8:**
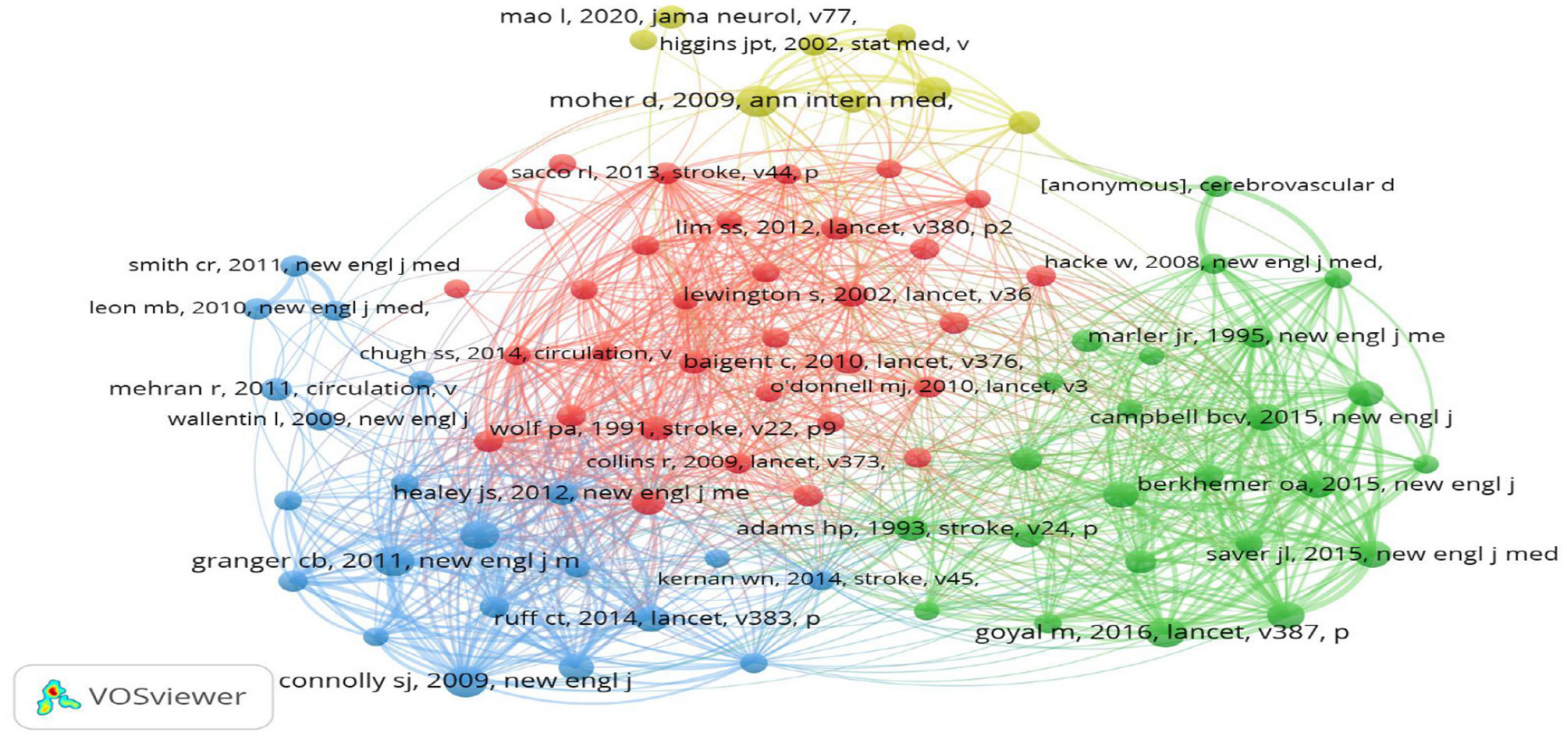
Co-cited references in stroke researches visualization.

Over the past 10 years, 172,373 references have been cited in highly cited papers on stroke. To further explore the research dynamics and trends in this field, we identified 10 articles with the highest citation frequency (Table 3). In addition, 86 cited references were selected, and a co-citation network graph was generated with a threshold ≥30 (Figure 8). As shown in Figure 8, a positive co-citation relationship is observed among articles published in different years in the journals. There was one reference published in the New England Journal of Medicine in 2009 that has received the most citations: “Dabigatran vs. warfarin in patients with atrial fibrillation.” The results of this study demonstrate that anticoagulation with 110 mg dabigatran and warfarin is equivalent in patients with atrial fibrillation, with no significant difference in the risk of preventing stroke and systemic embolism. However, in patients receiving dabigatran, the risk of major bleeding was significantly lower than that in those receiving warfarin. When the dabigatran dose was increased to 150 mg, the risk of stroke and systemic embolism was significantly lower in the treatment group than that in the warfarin group, while the proportion of major bleeding remained stable (Connolly et al., 2009). This finding provides an important reference for anticoagulation therapy in patients with atrial fibrillation, showing the potential advantages of dabigatran. The lowest-cited reference was published in Lancet in 2014 (citation number: 63). The average citation value for the first 10 cited references was 70.7.

**Table 3:** Top 10 co-cited references in highly cited papers in the field of stroke research.

**Rank**	**Cited reference**	**Citations**
1	Connolly SJ, 2009, New Engl J Med, v361, p1139	85
2	Moher D, 2009, Ann Intern Med, v151, p264	84
3	Granger CB, 2011, New Engl J Med, v365, p981	78
4	Goyal M, 2016, Lancet, v387, p1723	76
5	Patel MR, 2011, New Engl J Med, v365, p883	74
6	Berkhemer OA, 2015, New Engl J Med, v372, p11	64
7	Goyal M, 2015, New Engl J Med, v372, p1019	63
8	Campbell BCV, 2015, New Engl J Med, v372, p1009	62
9	Saver JL, 2015, New Engl J Med, v372, p2285	61
10	Ruff CT, 2014, Lancet, v383, p955	60

### 3.7 Analysis of citation bursts

**Figure 9:**
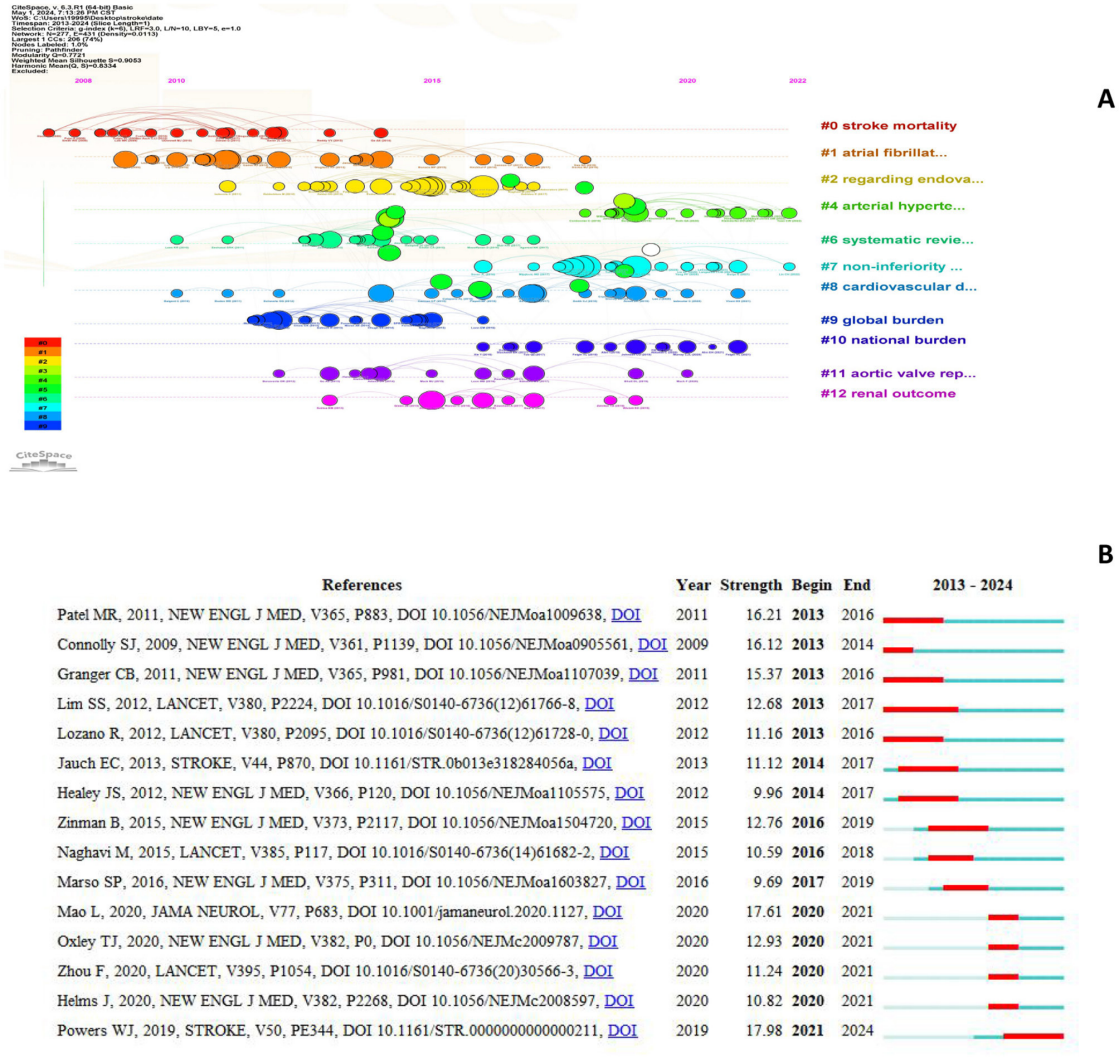
References was co-cited for clustering **(A)** and the top 15 references with the strongest citation bursts **(B)**.

The top 15 most-cited references are shown in Figure 9. A burst occurs when a publication receives a significantly higher number of citations than usual and lasts for at least 2 years (Jiang et al., 2022). The blue line represents the observation period from 2013 to 2024 and the red line indicates the burst time. The article “Guidelines for the early management of patients with acute ischemic stroke: 2019 update to the 2018 guidelines for the early management of acute ischemic stroke: a guideline for healthcare professionals from the American Heart Association/American Stroke Association,” published in the stroke, has the highest citation burst value (citation burst = 17.98) between 2013 and 2024. This is the latest citation outbreak in 2021 and has continued to date (Powers et al., 2019). The guidelines detail prehospital care, emergency evaluation, intravenous and intravenous treatment, and in-hospital management, including appropriately instituted secondary prevention measures within the first 2 weeks. The guidelines support the overall concept of a stroke care system and provide recommendations based on available evidence to guide physicians in the care of patients with acute arterial ischemic stroke. Additionally, the 2012 Lancet article “A comparative risk assessment of burden of disease and injury attributable to 67 risk factors and risk factor clusters in 21 regions, 1990–2010: a systematic analysis for the Global Burden of Disease Study 2010” cited the longest outbreak duration (=5 years). This study suggests that in 2010, the three leading risk factors for the global disease burden were high blood pressure, tobacco smoking, and household air pollution from solid fuels (Lim et al., 2012).

### 3.8 Frequency and clustering analysis of keywords

**Figure 10:**
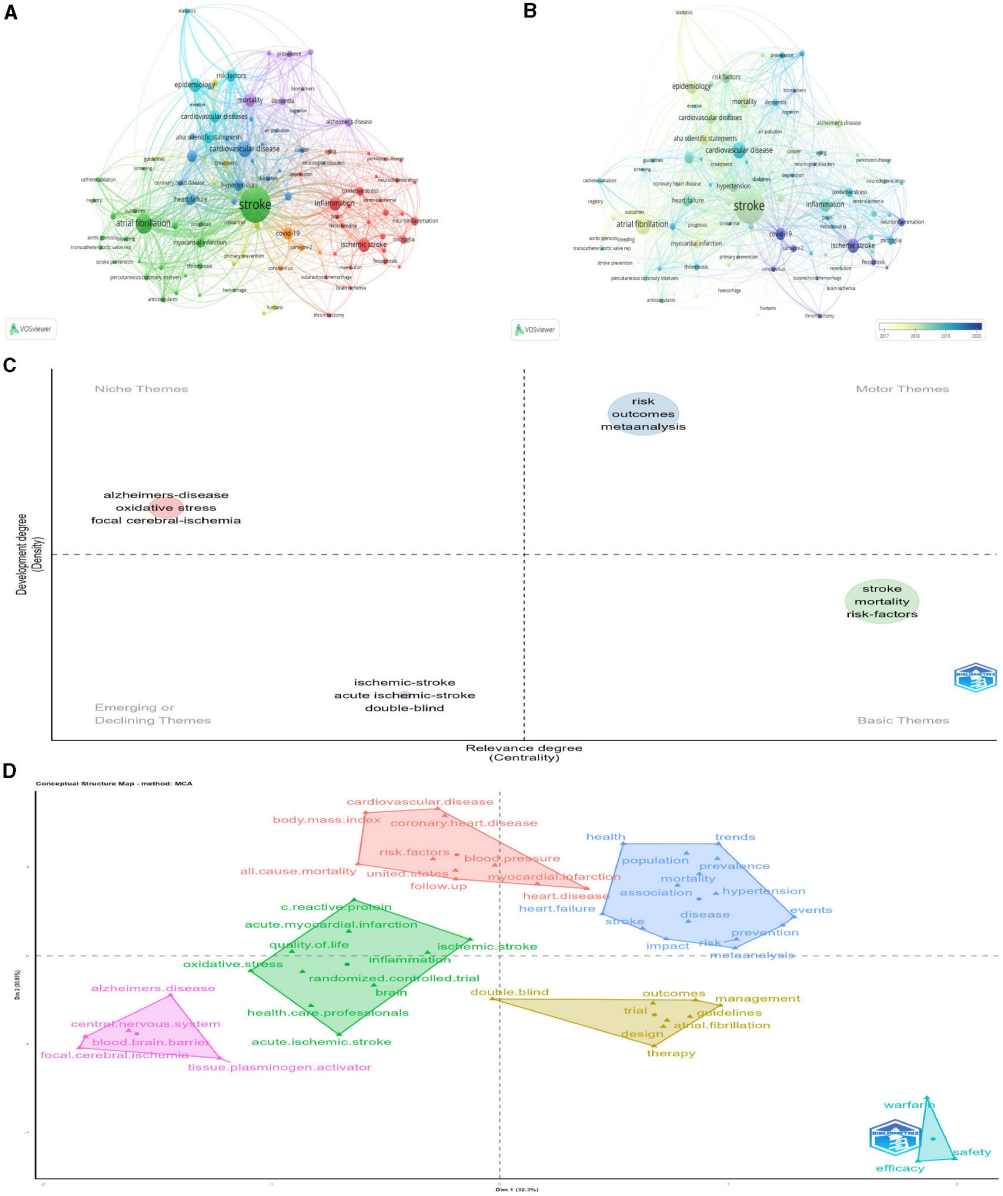
**(A)** Keyword co-occurrence network; **(B)** time-overlapping co-occurrence analysis network of keywords; **(C)** the topic map; **(D)** the topic classification.

Of the 3,625 keywords, 99 occurred at least 10 times and were analyzed further. If keywords had similar connotations, they were consolidated. Figure 10A illustrates the network visualization of these keywords, where the size of the nodes represents the keyword frequency and the proximity of the nodes signifies the strength of the relationships (Aria and Cuccurullo, 2017). Figure 10A presents a network visualization of these keywords. Group 1, depicted in green, concentrated on cardiocerebrovascular diseases linked to stroke, including atrial fibrillation, myocardial infarction, and “thrombosis.” Group 2, shown in blue, emphasized high-risk factors associated with cardiovascular diseases and meta-analyses, using keywords such as “cardiovascular disease,” “hypertension,” “obesity,” “diabetes,” and “meta-analysis.” The third light blue cluster primarily focused on epidemic risk factors associated with cardiovascular diseases, involving “cardiovascular disease,” “epidemiology,” “risk factors,” and “statistics.” Group 4 included the incidence, prevalence, and mortality rate of stroke-related diseases. Group 5, represented in red, centered on the pathogenesis of ischemic stroke with keywords such as “ischemic stroke,” “inflammation,” “oxidative stress,” and “neuroinflammation.” Figure 10B shows a visualization of the temporal overlapping of keywords.

To reflect critical themes in stroke research, a topic map and topic classification of keywords were created (Figures 10C, D). The keywords “risk,” “outcome,” “meta analysis” are motor themes, have high density and high centrality, which are important and developing well at present. The keywords “stroke,” “mortality,” “risk-factors” belong to basic theme, have high centrality but less dense, which represent important but not well-developed in the research field. The keywords including “Alzheimer's disease,” “oxidative stress,” and “focal cerebral ischemia” belong to niche theme, have high density and low centrality, which means that they are well-developed but not important to the current field. The keywords “ischemic-stroke,” “acute ischemic-stroke,” and “double-blind” belong to emerging theme, have high centrality and low density. Combined with overlay visualization, this research field is relatively marginal; however, there has been a trend of emergence and development in recent years.

### 3.9 Analysis of keywords bursts

**Figure 11:**
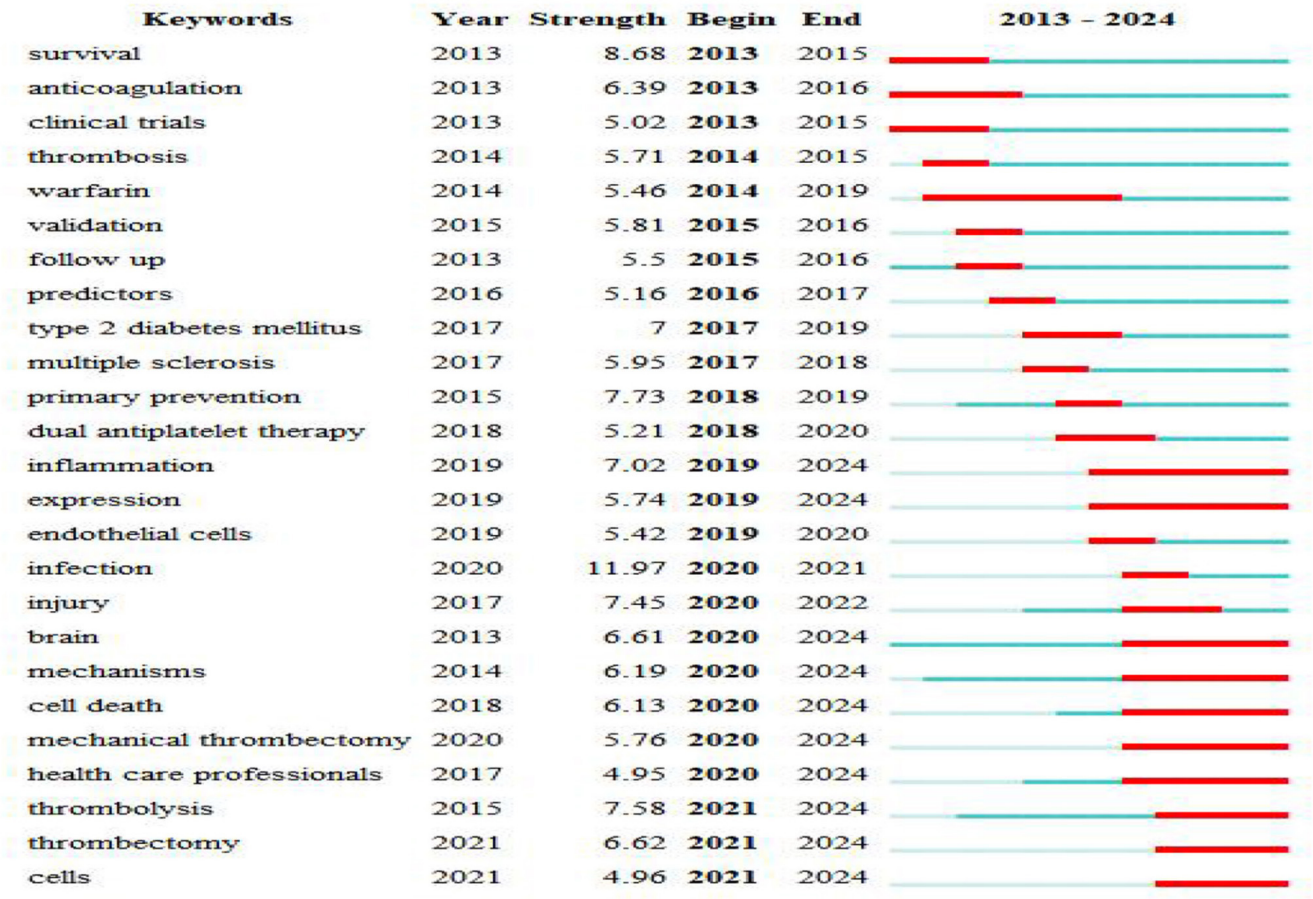
The top 25 keywords with the strongest citation bursts.

Figure 11 shows the 25 keywords with the highest citation bursts lasting for more than a year. The keywords “warfarin” (2014–2019), “inflammation” (2019–2024), and “expression” (2019–2024) have received the most consistent focus. Beyond “inflammation” “expression”, other keywords such as “brain” (2020–2024), “mechanisms” (2020–2024), “cell death” (2020–2024), “mechanical thrombectomy” (2020–2024), “health care professionals” (2020–2024), “thrombolysis” (2021–2024), “thrombectomy” (2021–2024), and “cells” (2020–2024) have also emerged recently. These findings suggest that future studies should focus on these areas.

## 4 Discussion

This study conducted a bibliometric review of 2,509 highly cited studies on stroke. The data indicate a consistent growth trend in the frequency of publications and the mean citation count for these pivotal works. From 2013 to 2023, the mean number of publications per annum among these influential articles was 227.73, with a corresponding annual citation average of 56.28. The gravity of stroke, as measured by its incidence, prevalence, case-fatality rate, and disability-adjusted life-years, underscores the enduring focus on stroke research by medical practitioners and public health professionals. Robust annual output within this scientific domain reflects this priority.

The top 10 nations, accounting for 82.66% of the referenced studies, are led by the United States, which contributes to a third (36.11%) of the total publications. The US also stands out for its international collaboration, leading seven of the top 10 slots in terms of collaborative frequency. These statistics underscore the US's preeminent role in global stroke research, a status likely attributable to its robust economic climate, significant investment in medical research, and prioritization of stroke studies. This field is poised to advance further through increased international scholarly collaboration, that is expected to enhance global research.

The top 10 academic institutions are largely distributed in the US, reflecting the country's prominent role in research output. While China leads in terms of the quantity of publications, none of its institutions are in the top 10. The UK holds the third position in terms of publications, with the University of Oxford coming in third with 116 papers in the top 10. Canada followed closely in fifth place, and the University of Toronto ranked fifth with 103 papers. Many collaborative studies have suggested that international partnerships are the key to enhancing research performance, particularly in resource-constrained environments.

Academic publishing relies heavily on peer-reviewed journals, which often conduct significant research within the field. Researchers can use the frequency of journal publications in the field of stroke to identify potential journals to submit their work. The New England Journal of Medicine tops the list with 223 publications, whereas Lancent has the highest impact factor (IF = 168.90), followed by the Journal of New England Journal of Medicine (IF = 158.50). Impact factor and journal citation reports (JCR) are standard metrics for assessing journal influence. JCR categorizes journals into quartiles (Q1–Q4) based on their IF, and all the top 10 journals by the number of papers are in the Q1 category. Furthermore, the top 10 journals are all based in the US and the UK, with the US accounting for 70% and the UK accounting for 30%.

The objective of this study was to address the topic of research extensively investigated by scholars over a defined period. The number of citations is considered a metric of a publication's academic impact (Xu and Sun, 2020). Publications with a high number of citations tend to encapsulate the core issues within a given research domain. Identifying these hotspots involves analyzing citation frequencies and pinpointing works that are frequently referenced. In this instance, nine articles qualified as highly cited (over 4,000 citations) and strongly linked (over 10 connections). These top-tier articles were published between 2015 and 2019 and predominantly appeared in the New England Journal of Medicine (four), Circulation (three), Lancet (one), and Stroke (one).

Three articles published in Circulation (Benjamin et al., 2017, 2018, 2019), penned by the American Heart Association Council on Epidemiology and Prevention Statistics Committee and Stroke Statistics Subcommittee, collated the latest Figures on heart disease, stroke, and cardiovascular risk factors used in the AHA My Life Check. These articles were updated in 2017, 2018, and 2019 and were entitled “Heart Disease and Stroke Statistics.” The 2017 update emphasized the advantages of substantial blood pressure (BP) reduction in clinical trials (Benjamin et al., 2017), which reduced the risk of stroke outcomes. It also highlighted that adherence to a Mediterranean diet abundant in nuts and olive oil was associated with a lower risk of stroke. The 2018 update pointed out that there were significant racial and regional disparities in stroke risks and outcomes, with the impact of hypertension management on stroke risk being more significant in those receiving intense treatment (Benjamin et al., 2018). The 2019 update found that although age-standardized mortality rates for ischemic and hemorrhagic stroke worldwide decreased from 1990 to 2015, the actual number of annual stroke cases, related deaths, and disability-adjusted life years increased. The majority of the global stroke burden falls in low- and middle-income countries (Benjamin et al., 2019).

Four key studies were published in the New England Journal of Medicine, in 2015. Goyal et al. (2015) suggested that urgent endovascular treatment could improve functional recovery and reduce death rates in patients with acute ischemic stroke caused by a blocked main artery, limited brain damage, or sufficient blood flow through alternative routes. Campbell et al. (2015) demonstrated the advantages of early blood clot retrieval using the Solitaire FR stent retriever system, as opposed to using the clot-dissolving drug alteplase alone, in patients with ischemic stroke and signs of salvageable brain tissue on CT perfusion scans. Furthermore, Berkhemer et al. (2015) and his associates confirmed the effectiveness and safety of intra-arterial treatment within the first 6 h after stroke in patients with blockage of the main brain artery in the frontal circulation. Lastly, Sarafidis and Tsapas (Sarafidis and Tsapas, 2016) reported that patients with type 2 diabetes and were at high risk of cardiovascular issues who were treated with empagliflozin experienced a lower incidence of cardiovascular events and all-cause mortality when the medication was included in their standard care.

The 2016 findings from Goyal et al. (2016), published in The Lancet, indicated that endovascular thrombectomy can be advantageous for the majority of patients experiencing acute ischemic stroke due to blockage in the anterior circulation, regardless of individual patient traits or regional location. This has contributed to a shift in the treatment paradigm for acute ischemic stroke, which is caused by clot-blocking of blood vessels in the brain. Endovascular thrombectomy is a minimally invasive procedure that involves removal of the clot from the affected blood vessel using a catheter threaded through the arteries to the site of blockage.

In 2018, Powers et al. from the American Heart Association/American Stroke Association (Powers et al., 2019) Published A Guideline for Healthcare Professionals, Guidelines for the Early Management of Patients With Acute Ischemic Stroke, which are based on the best evidence currently available, guidelines detailing prehospital care, urgent and emergency evaluation and treatment with intravenous and intra-arterial therapies, and in-hospital management, including secondary prevention measures that are appropriately instituted within the first 2 weeks. These guidelines support the overarching concept of stroke care systems in both pre-hospital and hospital settings.

Because keywords affect the core content of a study, co-occurrence analysis can identify high frequency keywords that appear in different studies, thus helping researchers to quickly grasp research hotspots. The most frequently used keywords were “risk,” “stroke,” and “mortality.” The mean “stroke” frequency was 255. From the topic map and subject word classification results, we know that mortality and risk factors of stroke, as basic themes, are an important part of the research field; however, further research is required. Meta-analysis of stroke risk and outcome is a motor theme, has been performed extensively in this field and is relatively mature, and some related studies have been conducted on “Alzheimer's disease,” “oxidative stress,” and “focal cerebral ischemia,” but the subject area is less central, so the development in the subject field is currently less important. “Ischemic stroke,” “acute Ischemic stroke,” and “double-blind experiment” belong to emerging themes, because ischemic stroke has the highest incidence of all stroke types, and is expected to be one of the important research topics in this field in the future.

In the factor analysis, the top 50 keywords were divided into six major categories, with four major categories related to the center. The first classified topics are: fatal related factors, meta-analysis, mortality mechanism, epidemic trend of event occurrence, prevention strategies and other related topics. The second major category includes: risk factors for stroke and various causes of death, including cardiovascular disease, hypertension, and body mass index. The third category of topics was mainly divided into: ischemic stroke and acute ischemic stroke inflammation, acute myocardial infarction, randomized controlled trials, health care, and quality of life. These keywords are classified re?ect the core content of the stroke study.

The CiteSpace “burst detection” method identifies keywords or cited references with significant changes over time (Chen, 2006). Researchers can use keywords and cited references with burst features to explore hotspots. In this study, “inflammation,” “expression,” “mechanisms, “thrombolysis,” “thrombectomy,” and “cell” etc. were keywords that continued to burst as of 2024. This suggests that the pathogenesis of stroke, thrombolysis, and thrombectomy is a future research hotspot. In addition, one cited reference will continue to burst by 2024. The guidelines in this literature provide general recommendations based on currently available evidence to guide clinicians caring for adult patients with acute arterial ischemic stroke.

## 5 Conclusions

This article conducted a bibliometric analysis of 2,509 highly cited stroke research papers, revealing the current status and development trends in this field. The following is an in-depth exploration of the analysis results:

### 5.1 Research hotspots and future directions

Inflammation and thrombolytic therapy: the persistent burst of keywords such as “inflammation” and “thrombolysis” indicates that the pathogenesis of stroke, thrombolytic therapy, and thrombectomy will be the focus of future research. This suggests the need to further investigate the role of inflammation in the onset and development of stroke and develop more effective thrombolytic therapy strategies. Mechanism research: the burst of keywords such as “mechanism” and “cell” indicates that stroke researchers are increasingly focusing on studying the pathogenesis. This study provides a theoretical basis for the development of new therapeutic drugs and preventive strategies. Neuroimaging: the emergence of keywords such as “imaging” and “CT scan” suggests that neuroimaging techniques are becoming increasingly widely used in the diagnosis, evaluation, and treatment of stroke. In the future, it will be necessary to further develop and apply new imaging techniques to assess the pathophysiological changes and treatment effects of stroke more accurately. Personalized treatment: the emergence of keywords such as “gene” and “epigenetics” indicates that personalized treatment will become an important direction in stroke treatment. Further research is needed to investigate the role of genes and epigenetics in the pathogenesis of stroke to develop more precise treatment plans.

### 5.2 National and regional cooperation

The leading position of the United States in the field of stroke research is undeniable, with research output and influence ranking first globally. China ranks second in terms of research output; however, its research influence still needs to be improved, and international cooperation is an important way to improve the level of stroke research. There is extensive cooperation between developed countries, such as the United States, the United Kingdom, Germany, and Canada. In the future, it will be necessary to strengthen cooperation with other countries, especially developing countries, to jointly promote stroke research.

### 5.3 Research institutions and journals

Institutions such as Harvard Medical School and Brigham and Women's Hospital have made outstanding contributions to the field of stroke research, and their research results are of great significance in promoting the development of this field. Top journals such as the New England Journal of Medicine and The Lancet have published a large number of high-quality stroke research papers, playing an important role in promoting the development of this field.

### 5.4 Researcher influence

Researchers such as Yusuf S have made significant contributions to the field of stroke research, and their research results are of great significance in promoting the development of this field. In the future, more excellent stroke researchers need to be trained to promote the development of this field.

Stroke research is a field full of challenges and opportunities. In the future, we need to strengthen international cooperation, focus on research hotspots, and train outstanding stroke researchers to promote the development of this field and to make greater contributions to human health.

## 6 Strengths and limitation

This study had some limitations. First, it exclusively incorporates extensively highly cited English-language articles indexed in the WoSCC repository. Although WoSCC encapsulates the most premium research, it can potentially skew our findings. Second, the inclusion of recently released premium research may be compromised by a time lag in citations, necessitating future updates. Nonetheless, this study provides substantial aid to researchers in the field by offering insights into the progression, focal points, trends, and cutting-edge developments in stroke research as well as highlighting areas calling for additional investigation.

## References

[B1] Aria M. Cuccurullo C. (2017). bibliometrix: an R-tool for comprehensive science mapping analysis. J. Inform. 11, 959–975. 10.1016/j.joi.2017.08.007

[B2] Benjamin E. J. Blaha M. J. Chiuve S. E. Cushman M. Das S. R. Deo R.. (2017). American Heart Association Statistics Committee and stroke statistics subcommittee. heart disease and stroke statistics-2017 update: a report from the American Heart Association. Circulation 135, e146–e603. 10.1161/CIR.0000000000000491 28122885 PMC5408160

[B3] Benjamin E. J. Muntner P. Alonso A. Bittencourt M. S. Callaway C. W. Carson A. P.. (2019). American Heart Association Council on Epidemiology and Prevention Statistics Committee and Stroke Statistics Subcommittee. Heart Disease and Stroke Statistics-2019 update: a report from the American Heart Association. Circulation 139, e56–e528. 10.1161/CIR.0000000000000659 30700139

[B4] Benjamin E. J. Virani S. S. Callaway C. W. Chamberlain A. M. Chang A. R. Cheng S.. (2018). American Heart Association Council on Epidemiology and Prevention Statistics Committee and Stroke Statistics Subcommittee. Heart Disease and Stroke Statistics-2018 update: a report from the American Heart Association. Circulation 137, e67–e492. 10.1161/CIR.0000000000000573 29386200

[B5] Berkhemer O. A. Fransen P. S. Beumer D. van den Berg L. A. Lingsma H. F. Yoo A. J.. (2015). A randomized trial of intraarterial treatment for acute ischemic stroke. N. Engl. J. Med. 372, 11–20. 10.1056/NEJMoa1411587 25517348

[B6] Campbell B. C. Mitchell P. J. Kleinig T. J. Dewey H. M. Churilov L. Yassi N.. (2015). Endovascular therapy for ischemic stroke with perfusion-imaging selection. N. Engl. J. Med. 372, 1009–1018. 10.1056/NEJMoa1414792 25671797

[B7] Chen C. (2006). CiteSpace II: detecting and visualizing emerging trends and transient patterns in scientific literature. J. Am. Soc. Inform. Sci. Technol. 57, 359–77. 10.1002/asi.20317

[B8] Connolly S. J. Ezekowitz M. D. Yusuf S. Eikelboom J. Oldgren J. Parekh A.. (2009). Dabigatran versus warfarin in patients with atrial fibrillation. N. Engl. J. Med. 361, 1139–1151. 10.1056/NEJMoa0905561 19717844

[B9] GBD 2019 Stroke Collaborators (2021). Global, regional, and national burden of stroke and its risk factors, 1990-2019: a systematic analysis for the Global Burden of Disease Study 2019. Lancet Neurol. 20, 795–820. 10.1016/S1474-4422(21)00252-0 34487721 PMC8443449

[B10] Goyal M. Demchuk A. M. Menon B. K. Eesa M. Rempel J. L. Thornton J.. (2015). Randomized assessment of rapid endovascular treatment of ischemic stroke. N. Engl. J. Med. 372, 1019–1030. 10.1056/NEJMoa1414905 25671798

[B11] Goyal M. Menon B. K. van Zwam W. H. Dippel D. W. Mitchell P. J. Demchuk A. M.. (2016). Endovascular thrombectomy after large-vessel ischaemic stroke: a meta-analysis of individual patient data from five randomised trials. Lancet 387, 1723–1731. 10.1016/S0140-6736(16)00163-X 26898852

[B12] Hicks D. Wouters P. Waltman L. de Rijcke S. Rafols I. (2015). Bibliometrics: the Leiden Manifesto for research metrics. Nature 520, 429–431. 10.1038/520429a 25903611

[B13] Ihaka R. Gentleman R. (1996). R: a language for data analysis and graphics. J. Comput. Graph. Stat. 5, 299–314. 10.1080/10618600.1996.10474713

[B14] Jiang S. Liu Y. Zheng H. Zhang L. Zhao H. Sang X.. (2023). Evolutionary patterns and research frontiers in neoadjuvant immunotherapy: a bibliometric analysis. Int. J. Surg. 109, 2774–2783. 10.1097/JS9.0000000000000492 37216225 PMC10498839

[B15] Jiang S. T. Liu Y. G. Zhang L. Sang X. T. Xu Y. Y. Lu X.. (2022). Immune-related adverse events: a bibliometric analysis. Front. Immunol. 13:7703. 10.3389/fimmu.2022.1096806 36591239 PMC9797501

[B16] Lim S. S. Vos T. Flaxman A. D. Danaei G. Shibuya K. Adair-Rohani H.. (2012). A comparative risk assessment of burden of disease and injury attributable to 67 risk factors and risk factor clusters in 21 regions, 1990-2010: a systematic analysis for the Global Burden of Disease Study 2010. Lancet 380, 2224–2260. 10.1016/S0140-6736(12)61766-8 23245609 PMC4156511

[B17] Mukherjee D. Lim W. M. Kumar S. Donthu N. (2022). Guidelines for advancing theory and practice through bibliometric research. J. Bus. Res. 148, 101–115. 10.1016/j.jbusres.2022.04.042

[B18] Powers W. J. Rabinstein A. A. Ackerson T. Adeoye O. M. Bambakidis N. C. Becker K.. (2019). Guidelines for the early management of patients with acute ischemic stroke: 2019 update to the 2018 guidelines for the early management of acute ischemic stroke: a guideline for healthcare professionals from the American Heart Association/American Stroke Association. Stroke 50, e344–e418. 10.1161/STR.0000000000000211 31662037

[B19] Sarafidis P. A. Tsapas A. (2016). Empagliflozin, cardiovascular outcomes, and mortality in type 2 diabetes. N. Engl. J. Med. 374:1092. 10.1056/NEJMc1600827 26981941

[B20] Van Eck N. Waltman L. (2010). Software survey: VOSviewer, a computer pro- gram for bibliometric mapping. Scientometrics 84, 523–538. 10.1007/s11192-009-0146-3 20585380 PMC2883932

[B21] Xu A.-H. Sun Y.-X. (2020). Research hotspots and effectiveness of repetitive transcranial magnetic stimulation in stroke rehabilitation. Neural. Regen. Res. 15:2089. 10.4103/1673-5374.282269 32394967 PMC7716019

[B22] Yeung A. W. K. (2019). Comparison between scopus, web of science, PubMed and publishers formislabelled review papers. Curr. Sci. 116, 1909–1914. 10.18520/cs/v116/i11/1909-1914

